# Perception of Patient Safety Culture Among Healthcare Practitioners in Dammam and Jeddah, Saudi Arabia

**DOI:** 10.3390/healthcare14060767

**Published:** 2026-03-18

**Authors:** Amani K. Alanazi, Mahmoud M. Berekaa, Abdulmalik S. Alsaif, Khalid S. Alsahli, Mohammed T. Aljassim, Mohammed A. Al-Warthan, Manna M. Alwadei

**Affiliations:** Department of Environmental Health, College of Public Health, Imam Abdulrahman Bin Faisal University (IAU), P.O. Box 1982, Dammam 31441, Saudi Arabia; amani919kh@gmail.com (A.K.A.); aalsaif@iau.edu.sa (A.S.A.); ksalsahli@iau.edu.sa (K.S.A.); mtaljassim@iau.edu.sa (M.T.A.); malwadei@iau.edu.sa (M.M.A.)

**Keywords:** patient safety culture (PSC), AHRQ, HSOPSC, error reporting, incidence reporting, Dammam, Jeddah, Saudi Arabia

## Abstract

**Background:** There is escalating concern about patient safety among healthcare workers (HCWs) due to the alarming number of deaths and disabilities. Objective: The main aim of this study is to explore the perceptions of patient safety culture (PSC) among HCWs in two major cities in Saudi Arabia, compare the findings with those of international studies, and highlight the major strengths and challenges that affect the incorporation of PSC in these two cities. **Methods:** A cross-sectional design was used to assess PSC among HCWs in hospitals in Dammam and Jeddah, Saudi Arabia. This study utilised the Saudi Hospital Survey on Patient Safety Culture (HSPSC), which is commonly used by HCWs in the Ministry of Health, and the results were compared with those from the Agency for Healthcare Research and Quality (AHRQ). Data were analysed using the Statistical Package for the Social Sciences (SPSS). Chi-square tests were used to assess the association between patient safety ratings and the reporting of patient safety events. An independent t-test was used to examine differences in mean scores of study variables between the two cities. A *p*-value of less than 0.05 was considered statistically significant. **Results:** Out of 737 participants, 357 completed the survey. Physicians were the most common (27%), followed by nurses (11.9%), in Dammam. In Jeddah, nurses were first (20%), followed by transporters and security (12.7 and 11.6%, respectively). Overall, error reporting and supervisor support were areas of strength, while management commitment, teamwork, and incident reporting were identified as areas needing improvement. More than 94% of employees rated patient safety positively. **Conclusions:** This study highlights the importance of HCWs’ perceptions of PSC in Dammam and Jeddah. Overall, patient safety ratings among participants were highly positive (94%), reflecting favourable views of teamwork, supervisor support, and hospital management’s commitment. Although event reporting and teamwork were recognised as major strengths, it is still necessary to implement regular patient safety training programmes and formal patient safety policies to address existing gaps. Overall, PSC ratings were more positive and significantly higher in Dammam than in Jeddah. These findings may help policymakers and managers enhance patient safety and develop more robust systems to protect both patients and HCWs.

## 1. Introduction

The issue of patient safety has garnered increasing attention due to reports issued by the World Health Organization, highlighting alarming statistics. Medical errors are the third leading cause of death annually in the US [1]. Resource-limited settings face greater challenges, with 134 million adverse events and more than 2.6 million deaths annually [2,3]. Patient safety culture (PSC) is considered one of the top ten causes of death and disability globally. PSC involves the collective attitudes, behaviours, and practices that determine how healthcare organisations prioritise and manage patient safety; thus, it is considered a factor that potentially reduces healthcare-related harm to a tolerable level. Furthermore, unsafe healthcare procedures are recognised as one of the leading causes of infection transmission and harm [4,5]. Approximately 5% of healthcare representatives make diagnostic errors in healthcare, which have serious ramifications. Statistics indicate that an individual is exposed to one diagnostic error during their life [4].

Approximately 10% of individuals experience an injury while receiving medical care, and more than three million deaths are attributed to inferior healthcare annually. In addition, approximately 4% of deaths reported in low- to middle-income countries are associated with poor medical care [6]. According to Slawomirski [7], as many as four out of ten patients may suffer an injury in primary and ambulatory settings, but up to 80% (23.6–85%) of these injuries could have been prevented. Adverse events, including misidentification, diagnostic and medication errors, hazardous surgical procedures, patient falls, and unsafe blood transfusions, can be a major cause of patient injury [4]. Patient injuries can decrease global economic growth by 0.7% annually. The annual indirect cost of harm is estimated to be trillions of US dollars on a global basis [6]. Patient safety is defined as the absence of preventable injury to a patient and the acceptable minimisation of the risk of needless harm related to medical care. According to the World Health Organization (WHO) [4], it is a framework of structured activities that, when considered within the larger context of the health system, generates beliefs, procedures, performances, skills, and environments in the healthcare setting that consistently and sustainably lower risks, reduce the probability of preventable injury, decrease the probability of error, and lessen the impact of injury when it occurs. The positive aspects of PSC include open communication about safety issues, productive cooperation, and management commitment to patient safety. It also involves sharing values, attitudes, and standards among staff members [8,9,10].

Patient safety culture is a complex phenomenon that is constantly evolving in hospitals worldwide. Therefore, there is still an increasing amount of research fostering patient safety in Saudi Arabia, particularly in primary healthcare facilities. These facilities, which prioritise treatment, disease prevention, and improving quality of life, are widely regarded as the entry point to the healthcare system [11]. Various factors that contribute to a positive PSC, such as leadership and management, communication, information flow within and between units, and a non-punitive approach to incident reporting and error reporting through a structured mechanism for identifying, documenting, and analysing medical errors, adverse events, and near misses, have been extensively studied [12,13,14,15].

In Saudi Arabia, there is a significant interest in identifying the latest developments in the field of PSC in various primary health centres as well as governmental and private hospitals. However, healthcare representatives continue to face significant challenges in ensuring patient safety and establishing a strong work culture [16]. Several studies have revealed that healthcare facilities in Saudi Arabia are in need of improvements in various key areas, including organisational ethical accountability, lack of non-punitive mechanisms, unawareness of reporting policies, high workloads hindering timely reporting, insufficient training, low levels of comprehension, and reducing medical errors [17,18].

In 2017, the SPSC in Saudi Arabia launched the first cycle of a national project using the AHRQ HSOPSC survey tool version 1.0; since 2023, it has been in its 5th cycle, using version 2.0, with more than 170,000 surveys and 420 engaged hospitals from different healthcare sectors. International benchmarking of PSC domains (2019 to 2023) showed that the average percentage was 80% for positive response to teamwork, 47% for staffing and work pace, 76% for organisational learning and continuous improvement, 56% for response to error, 71% for supervisor or manager support for PS, 73% for communication about error, 65% for communication openness, 67% for reporting PS events, 63% for hospital management support for PS, and 69% for handoffs and information exchange. These results were comparable to international benchmarks but still need improvement in areas like management support, teamwork, and error response. Accordingly, the SPSC initiated a PSC improvement initiative that conducted workshops in six regions of the Kingdom over 2022–2023. These workshops resulted in a 9% increase in the positive response of the hospital’s management support in the 2023 reassessment phase [19].

Interestingly, for safer healthcare, the National Patient Safety Agency has created a safety circle based on the importance of reporting any event. This is the key step to understanding problems, finding sound solutions, avoiding serious consequences, and preventing their recurrence in the future [8,20,21]. Learning from mistakes made in the workplace and receiving criticism are two aspects of organisational learning. Leaders in patient care understand that substantial decreases in medical errors may only come about by enhancing collaboration within healthcare teams and creating a good work environment [22,23].

There is an urgent need to improve the quality of healthcare through a culture of patient safety and clinical effectiveness, with priority given to patients in university hospitals. Some of the main obstacles to providing quality healthcare are the poor levels of PSC, weak linguistic communication, and the lack of instructions provided to patients [24]. Consequently, regulatory entities ought to formulate strategies addressing these variables to foster a more robust patient safety environment [25]. A shift has recently occurred from relying on safety metrics such as accident rates, which measure historical safety events and past safety issues, to proactive approaches based on evaluating the current safety culture (leading indicators), such as measuring the safety climate [26].

Moreover, the Ministry of Health in Saudi Arabia [27] has employed accreditation as a strategy to increase the standard of healthcare services overall and enhance patient safety. Unfortunately, some healthcare institutions do not use accreditation as a tool to promote patient safety and quality. However, hospital accreditation may not always result in better outcomes; some research has found no meaningful connections between patient satisfaction and hospital recognition [28].

The current study aimed to assess the perception of PSC among HCWs in the cities of Dammam and Jeddah, using the Saudi Hospital Survey on Patient Safety Culture (HSOPSC). This study closely examined the shared beliefs, values, and behaviours about patient safety among HCWs in both cities. Emphasis was placed on benchmarking between hospitals in Dammam and Jeddah, as well as against the Agency for Healthcare Research and Quality (AHRQ), to highlight the major differences and areas for improvement. Moreover, the study aimed to provide recommendations to adopt patient safety policies and help policymakers and managers enhance patient safety and create more robust systems to protect patients and workers.

## 2. Materials and Methods

### 2.1. Study Location

The study was conducted among HCWs in four governmental hospitals providing specialised tertiary care, with a total of 1200 beds and approximately 5000 employees, over three months (January to March 2024) in two major cities of Saudi Arabia, Dammam and Jeddah. Jeddah, the second major city bordering the Red Sea in western Saudi Arabia, is the primary gateway to Mecca and serves as the central pillar of the Kingdom’s economic diversification strategy under Vision 2030. Jeddah’s metropolitan population is estimated to be between approximately 5.1 million and 5.9 million as of January 2026 [29,30]. Dammam city is Saudi Arabia’s third-largest urban hub and the administrative and economic centre of the Eastern Province. Comprising Dammam, Al-Khobar, and Dhahran, the region spans approximately 3900 km^2^ with a current population of around 2 million and is driven by the petroleum industry [31,32,33].

### 2.2. Research Design and Data Collection

In this research, a cross-sectional survey was employed to evaluate the extent to which HCWs recognise shared beliefs, values, and behaviours about patient safety culture, and their commitment to fostering it. The required sample size for the study was calculated to be 357 participants from a target population of 5000, using the standard formula for proportion estimation in health studies (the proportion estimate is 50%, 95% confidence level, and 0.05 precision) [34]

Sample Size = [z^2^ ∗ p(1 − p)]/e^2^/1 + [z^2^ ∗ p(1 − p)]/e^2^ ∗ N]

N = population size.
z = z-score.
e = margin of error.
p = standard deviation.

In the current study, the Hospital Survey of PSC (HSOPSC) version 2.0 was used. This survey was designed for the purpose of evaluating PSC and has been utilised in numerous countries, including Saudi Arabia and several countries in Asia and Europe [35]. In fact, the HSPSC in Saudi Arabia is a specific, adapted implementation of the standardised HSOPSC version 2.0 that was developed and validated by the US Agency for Healthcare Research and Quality (AHRQ) framework. The major adaptations include language, specific terminology adjustments, and the creation of a local comparative database. Indeed, this survey was managed at a national level by the Saudi Patient Safety Center (SPSC). The HSOPSC emphasises PSC as well as error and event reporting. It starts by raising questions regarding the demographic characteristics of the participants, followed by composite questions evaluating the PSC of participants. The questionnaire comprises 10 composite measures and 32 items. The 10 composites include patient safety teamwork, organisational learning and continuous improvement, communication about errors and communication openness, handoffs and information exchange, staff perceptions of supervisor support for patient safety, reporting patient safety events, hospital management commitment to patient safety, participants’ responses about error reporting in their unit/work area, staffing and work pace, and patient safety rating. Also, it includes questions recording the overall grade of patient safety in the work area/unit and the number of events reported over the past 12 months.

The survey targets HCWs, such as physicians, practising and vocational nurses, psychologists, respiratory therapists, technicians, supervisors, managers, clinical leaders, and directors. The survey was administered in English through several communication channels, including E-mail, WhatsApp, and Google Form. The questionnaire was distributed to reach the target groups in the studied healthcare facilities in Dammam and Jeddah through each hospital’s public relations department, which has employees’ contact information.

### 2.3. Statistical Analysis

The data was analysed using SPSS 24.0 (SPSS Inc., Chicago, IL, USA), and descriptive statistics were used to summarise the participants’ responses to the questionnaire items in mean (−/+ SD) and frequency for both numerical and categorical variables. All the questionnaire items used a 5-point Likert scale. Some questions were negatively worded to improve the accuracy and quality of the data collected and assigned as (RI). For these questions, the scoring was reversed before analysis. The percentage of positive responses for each item was considered by adding up the positive responses (agree and strongly agree). Composite-level scores were calculated by summing the positive scores of the items within a composite scale and dividing by the number of items in that composite scale. The mean scores were then interpreted according to a classification system developed by Pimentel [36]. This system considers the equal interval between points on a 5-point scale (0.80) and defines the following categories: very low: 1.0 to less than 1.8; low: 1.8 to less than 2.6; medium: 2.6 to less than 3.4; high: 3.4 to less than 4.2; and very high: 4.2 to 5.0.

The Chi-square test was used to assess the association between the patient safety rating and the reporting of patient safety events. Before analysis, staff ratings of ‘Poor’ and ‘Fair’ were combined into a single category labelled ‘Poor’ (*n* = 22), which combines staff with potentially lower perceptions of patient safety. The remaining staff ratings (Good’, ‘Very Good, and ‘Excellent’) were grouped as ‘Good’ (*n* = 335), which combines staff with potentially higher perceptions of patient safety. An independent *T*-test was also used to illustrate the difference in the mean scores of the study variables across the two cities. For all tests, a *p*-value of less than 0.05 was utilised as the significance level.

### 2.4. Ethical Consideration

Approval from the Research Ethical Committee of Imam Abdulrahman bin Faisal University (IRB-PGS-2024-03-150) was acquired prior to data collection. Furthermore, each participant was required to provide written consent documenting their agreement and ability to participate in the study. Participants unwilling to complete the questionnaire had the right to leave the survey at any time. Non-HCWs in the healthcare facilities were excluded from participation in the study.

## 3. Results

### 3.1. Demographic Characteristics of Participants

Table 1 presents the job titles of the 357 HCWs who participated in the study. Physicians were the most common participants (50, 27%), followed by nurses (22, 11.9%) in Dammam. However, in Jeddah, nurses were the most common (34, 20%), followed by transporters and security (12.7% and 11.6%, respectively). Other participants included psychologists (28, or 7.8%) and respiratory therapists (31, or 8.7%) in Dammam. However, in Jeddah, office workers and the category combining pharmacists, physical therapists and respiratory therapists were included (12, 6.9% and 11, 6.4%). In both cities, the technician category had the lowest number of participants (0.5 and 1.7% for Dammam and Jeddah, respectively).

Regarding HCWs’ experience (Table 1), over half (52.2% and 50.31%) have worked at the hospital for six to ten years, and a significant portion (32.6% and 37.6%) have been there for one to five years in Dammam and Jeddah, respectively. In terms of unit tenure, the majority (61.4% and 51.4%) have been in their current unit for a similar period, and 21.2% and 31.2% have been working for 11 or more years, for participants in Dammam and Jeddah, respectively. For Dammam and Jeddah participants, approximately 65.2% and 68.8% typically work 30 to 40 h per week, while 20.1% and 19.1% work more than 40 h, respectively. Interestingly, for participants in Dammam and Jeddah, the vast majority (90.8% and 86.7%, respectively) of workers typically have direct contact with patients, while a small percentage (9.2% and 13.3%, respectively) do not have direct patient interaction.

A positive trend emerged with regard to patient safety reporting, with a significant proportion of participants (41.3% and 32.9%) reporting 6 or 10 patient safety events in the past year, while fewer participants (29.9% and 27.2%) reported three to five events in Dammam and Jeddah, respectively. A distinct group of 8 and 12 participants (4.3% and 6.9%) reported no patient safety events within the past year in Dammam and Jeddah, respectively.

### 3.2. Factors Affecting Patient Safety Culture Among Healthcare Workers

#### 3.2.1. Staff Perceptions of Patient Safety

**Figure 1:**
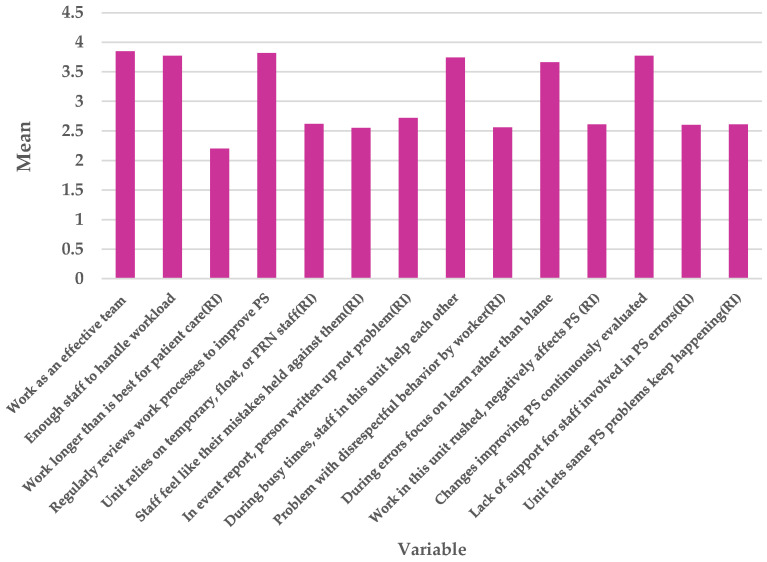
Staff perceptions of patient safety in work area (expressed as mean score).

Figure 1 presents staff perceptions of patient safety within a healthcare unit regarding four composites, including teamwork, work pace, and organisational learning and continuous improvement. Overall perceptions of patient safety (mean score of 3.08) were moderate. High scores on items related to effective teamwork and regular reviews of work processes (mean scores of 3.85 and 3.82, respectively) suggest a strong positive view of teamwork. In contrast, the lowest-scoring item—longer working hours in the unit than is optimal for patient care (reverse-scored; mean score of 2.20)—signals a potential concern. As presented in Figure 1, 60.8% of staff agreed or strongly agreed with this statement, suggesting that extended working hours may adversely affect patient safety.

#### 3.2.2. Staff Perceptions of Supervisor Support, Error Reporting, Event Reporting, and Hospital Management Commitment to Patient Safety

Healthcare workers reported a positive perception of supervisor support (mean score of 3.12 ± 0.91; medium level). Approximately 66.7% agreed or strongly agreed that supervisors act on reported patient safety concerns, consider staff suggestions, and value their input for improving patient safety (mean score of 3.46 ± 1.32). Conversely, only 18.5% of staff disagreed or strongly disagreed that supervisors did not wish staff to work faster, indicating that most staff perceive supervisors as prioritising speed over safety during demanding periods (Table 2).

Most HCWs reported a generally positive perception of error reporting (mean score of 3.41 ± 0.64; high level; Table 2). Transparency in error reporting was evident, with a high proportion of staff indicating they are informed about errors (mean score of 3.86 ± 1.19; high level). Open communication about safety was also positive. Staff felt comfortable speaking about potential patient safety issues, changes made based on reports, and even unsafe practices by superiors (mean scores of 3.74 ± 1.25; 3.64 ± 1.31, and 3.53 ± 1.34, respectively). However, there was less certainty about how receptive superiors are to concern and fear of asking questions (reversed item; mean scores of 3.20 ± 1.33 and 2.38 ± 1.21, respectively).

Interestingly, HCWs reported a generally positive perception regarding patient safety event reporting within the healthcare unit (score of 3.65 ± 0.97; Table 2). A high proportion of staff reported consistent reporting of events that reached the patient but did no harm (3.67 ± 1.34), which highlights a commitment to capturing all potential safety issues. Staff also indicated that they reported mistakes that were caught and corrected before reaching the patient (3.63 ± 1.14), which suggests a culture of proactive safety reporting.

In addition, HCWs’ perceptions of the hospital management’s commitment to patient safety were generally positive, with an overall mean score of 3.29 ± 0.79. Approximately 47.9% of participants agreed that hospital management provides adequate resources to improve patient safety, while 69.2% agreed or strongly agreed that hospital management demonstrates patient safety as a top priority. Furthermore, a majority (31.9%) strongly agreed that there was adequate time for exchanging patient care information during shift changes, with a mean score of 3.66 ± 1.33, while another 31.9% agreed. However, there were some areas where staff expressed less confidence. A significant proportion (38.1%) agreed or strongly agreed that management is only interested in safety after an adverse event (2.76 ± 1.32). Moreover, scores on reversed items regarding information sharing during patient transfers (2.80 ± 1.39) and shift changes (2.69 ± 1.41) suggest some concerns about complete and timely communication.

#### 3.2.3. Patient Safety Rating

**Figure 2:**
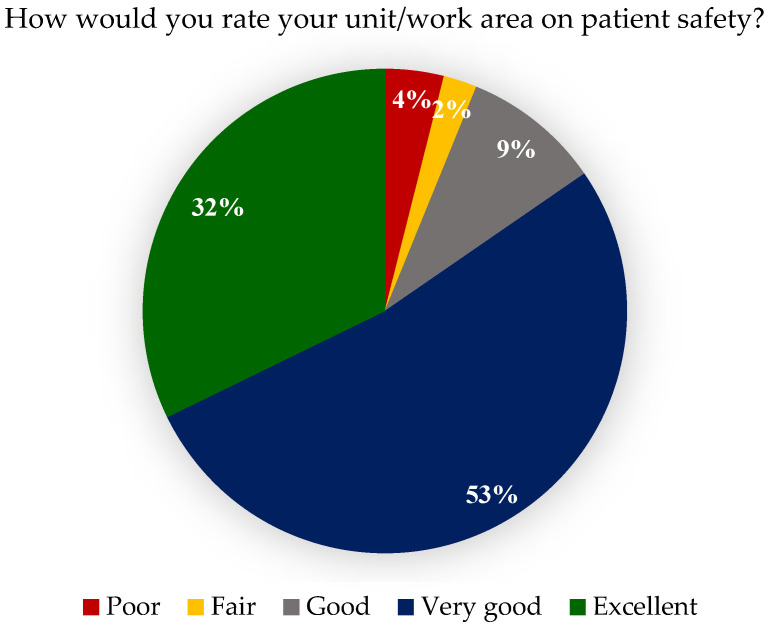
Participants’ responses about Patient Safety Ratings.

Interestingly, approximately 94% of staff rated patient safety positively, with a mean score of 4.07 ± 0.93, indicating a high level. This finding suggests that staff feel their unit or work area prioritises patient safety and maintains a positive safety culture (Figure 2).

#### 3.2.4. Association Between Patient Safety Rating and Event Reporting

The results presented in Table 3 reveal a significant association between patient safety rating and the number of events reported (*p*-value = 0.030 < 0.05). Safety events were more likely to be reported by those who rated patient safety on their unit as ‘Good’ (96%), as opposed to those who gave a rating of ‘Poor’ (6%). This suggests that staff who perceive higher levels of patient safety might report events differently from those who perceive lower levels. Moreover, staff who feel confident in their unit’s safety culture are more comfortable reporting even minor events; they may perceive a greater likelihood that their reports will be taken seriously.

#### 3.2.5. Comparing Patient Safety Culture in Dammam and Jeddah

The results presented in Table 4 reveal major differences in PSC among HCWs in the cities of Dammam and Jeddah. Supervisor support, communication openness, communication about error, event reporting and hospital management support for patient safety were significantly higher in Dammam than in Jeddah. Moreover, the patient safety rating was significantly higher in Dammam than in Jeddah (mean score of 4.29 ± 0.62 and 3.83 ± 1.12, respectively; *p*-value = 0.000). This indicates that healthcare providers in Dammam rated the overall PSC more positively. However, there was marginal significant difference in the mean score for patient safety teamwork between Dammam and Jeddah (*t* = 1.99, *p*-value = 0.047).

#### 3.2.6. Patient Safety Culture Benchmarking Between Dammam and Jeddah Hospitals and the Agency for Healthcare Research and Quality

**Figure 3:**
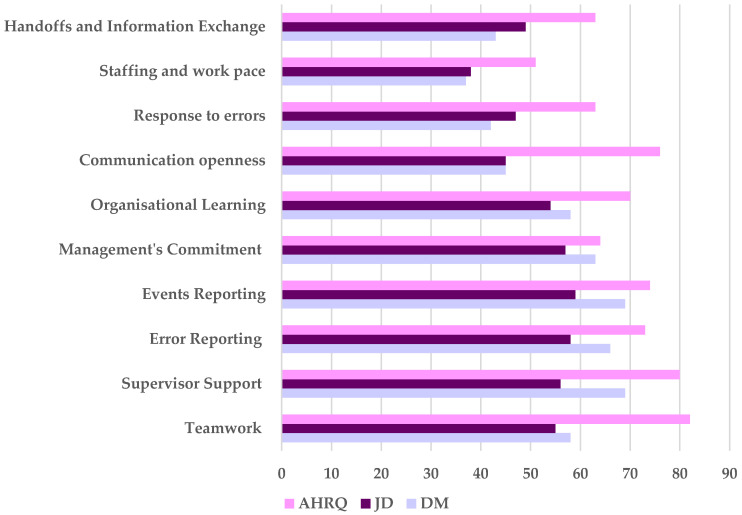
PSC composite measures benchmarking between Dammam (DM) and Jeddah (JD) hospitals and Agency for Healthcare Research and Quality (AHRQ) in the year 2024 [19].

The results presented in Figure 3 demonstrate that in both Dammam and Jeddah, HCWs’ responses on handoffs and information exchange (43% and 49%, respectively), staffing and work pace (37% and 38%, respectively), response to error (42% and 47%, respectively), communication openness (45% for both), supervisor support (69 and 56%, respectively), and teamwork (58% and 55%, respectively) were far below the AHRQ benchmark. While error reporting (66% and 58%, respectively), event reporting (69% and 58%, respectively), and management’s commitment (63% and 57%, respectively) were approaching the AHRQ benchmark.

Interestingly, the low positive score for teamwork is in line with the findings of [18,37] from the King Fahd Teaching Hospital (KFTH), which were far below AHRQ-2024 [19].

## 4. Discussion

### 4.1. Staff Perceptions of Teamwork and Patient Safety

This study aimed to reveal the perceptions of PSC among HCWs in healthcare facilities in Dammam and Jeddah, Saudi Arabia. Staff perceptions of teamwork and patient safety within the healthcare unit indicated a moderate response level with room for improvement, while still reflecting a generally positive view of teamwork effectiveness. These findings are consistent with a study conducted in healthcare centres in the Eastern Region [18], which identified teamwork, information exchange, and care delivery processes as key strengths. Strengthening safety culture initiatives may further help reduce the occurrence of adverse events and medical errors [38]. Although this study did not directly examine the relationship between teamwork and medical errors, previous research has highlighted the important role of effective teamwork and communication in improving patient safety and reducing clinical errors [39]. Similarly, other studies [40,41,42] reported that nurses perceive effective teamwork as essential for achieving patient safety outcomes in clinical units. Strengthening teamwork within healthcare units may therefore contribute to a stronger patient safety culture and improved patient outcomes. Moreover, Costello et al. [43] revealed that improvements in communication and teamwork play a crucial role in reducing medical and nursing errors, ultimately leading to better patient safety and improved healthcare.

In contrast, Al Abdulmalik et al. [44] assessed the PSC at the Primary Healthcare Corporation (PHCC) in Qatar utilising the AHRQ HSOPSC. The study identified teamwork, error reporting, management support, communication, non-punitive response to errors, and staffing as major areas of strength. Consistent with the results of the present study, another study by [45] revealed that implementing communication and teamwork strategies, such as obtaining clinical and administrative assistance at all levels of management, supporting staff attendance at presentations, and promoting further learning opportunities within units, can improve PSC.

### 4.2. Staff Perceptions of Supervisor Support, Error Reporting, Event Reporting and Hospital Management’s Commitment to Patient Safety

The present study has revealed a strong culture of reporting patient safety events within the unit, confirming comfortable reporting of incidents and near misses. This finding is consistent with the existing literature on the importance of reporting incidents and maintaining safe practices. A recent study by Almansour [46] highlighted the reporting system as a major barrier hindering incident and near-miss reporting by HCWs in Saudi Arabia. Braiki [47] discussed the major factors influencing medication errors and near misses among nurses in a systematic review study, revealing that errors and near misses were associated with perceived behavioural control, subjective norm and attitude. Recently, Augustine [48] reported the impact of electronic incidence reporting technology in improving patient safety. HCWs studied by [49] support the value of error reports as information resources for developing preventive measures aimed at reducing the number of pharmaceutical errors.

In contrast, according to the annual report issued by the Saudi Patient Safety Center [50], approximately 50% of health practitioners did not report any incidents related to patient safety in the health unit, a percentage that has remained the same for the past four years. In addition, lower rates of adverse events were not observed in studies with higher PSC ratings. Previous research on the relationship between PSC and quality outcomes supports this finding [51,52].

Many investigations have revealed the absence of a strong PSC due to several problems with reporting. These include a lack of punitive procedures, a heavy workload that makes it difficult to report on time, a lack of knowledge, a lack of comprehension, and a lack of awareness about reporting policies. According to [17], many healthcare facilities in Saudi Arabia lack anonymous reporting methods. Moreover, Alkubati et al. [53] reported a moderate to high level of overall perception of patient safety with low positive response rates in non-punitive response to errors (38%). In addition, error reporting and non-punitive response to errors and staffing were identified as major areas of strength that support PSC in Qatar hospitals [44]. At the international level, Alabdullah et al. [12] reported that HCWs raised concerns about a punitive environment that discourages error reporting. Moreover, Fuseini et al. [54] demonstrated that non-punitive responses to errors were the lowest rated and not considered a high priority, falling below the benchmark set by the AHRQ, as recorded by the HSPSC data collection tool.

The present study revealed that most employees (66.7%) agreed or strongly agreed that supervisors should act on reported concerns about patient safety. According to [55,56], HCWs are hesitant to disclose unfavourable incidents to their supervisors and are more inclined to report the occurrence to a colleague. While reporting an incident to a senior staff member—especially among physicians—is rare, it is more common when the occurrence indicates a procedural violation that has a negative consequence [57]. It was found that a vital component of preserving high-quality treatment is learning from errors. Moreover, using administrative records, incidence rates were discovered in 38% of the investigations. According to [58], registers may be a helpful technique because they allow for higher sample sizes and unit-level data extraction. Recently, Greenspan and Payne [59] emphasised the role of supervisors, not only in providing patient safety within the constraints of sustainability but also in ensuring a safe environment.

According to the results of this study, PSC is more appreciated in health units that follow a comprehensive error reporting strategy (High level = 3.41) that prioritises systematic learning over individual blame. Moreover, managers play a critical role in the success of an error reporting plan in the health sector because they oversee the smooth operation of healthcare facilities and improve reporting. Birk et al. [60] advise that planning, organising, leading, and regulating are the four fundamental tasks of a manager that help them succeed in their role within a company. Establishing a culture of patient safety through incident reporting requires removing three important factors: blame, fear, and silence [15,18].

Furthermore, hospital administrators find PSC to be a complicated issue that is challenging to operationalise. A strong PSC can be predicted by several factors, including the consistent commitment of management and leadership, communication, the flow of information within and between units, and a shared understanding of the importance of patient safety [61]. This is largely consistent with the present study’s findings that employees feel that supervisors generally accept patient safety concerns and value employee input. In addition, this reflects open communication between them to continuously improve PSC. Similarly, leadership plays a crucial role in fostering teamwork, being the key pillar for success in hospitals through enhancing the communication between various departments and levels of staff, thus improving PSC [62,63]. Recently, Alshareef [64] reported that the significant predictors of patient safety are teamwork, work pressure, and hospital management support in three private hospitals in Jeddah, Saudi Arabia. Positive patient safety results in East Asian hospitals were most closely linked to safety culture elements such as feedback and communication, reporting occurrences often, teamwork across units, and managers’ support for patient safety [65,66,67].

The results of the present study also indicated that employees’ opinions about the hospital administration’s dedication to patient safety were largely positive. Approximately 47.9% of the sample indicated that the hospital administration provided sufficient funding to enhance patient safety. Meanwhile, more than two-thirds of employees (30.5% and 38.7%) believed that patient safety is a major priority when it comes to hospital management operations. Therefore, the relevant authorities should endeavour to develop policies that address these factors, thereby positively impacting the culture of patient safety, especially by learning from mistakes and avoiding repeating them in the future [25].

This study reveals that healthcare providers in Dammam receive support from their supervisors and that there is a significantly higher administrative commitment in hospitals, which is positively reflected in their PSC. This is consistent with a study conducted on nursing staff who have a professional commitment, which revealed that they have a high PSC [68]. According to [69], patient safety can be enhanced, and nursing staff performance and skills can be improved through continuous observation and alerts by supervisors and management.

The results of this study indicated that healthcare providers in Dammam were more likely to report adverse events compared to Jeddah, which made their PSC more positive. According to some studies, increasing reported events can contribute to reducing medication errors and improving patients’ immunity [70].

Overall, the findings of this study indicate that hospital staff believe patient safety is the highest priority for hospital management and that adequate resources are provided. However, there is room for improvement, especially in communication during shifts and a marked tendency to react to safety issues after they occur rather than proactively prevent them. Therefore, managers must genuinely commit to patient safety and set a good example for others to follow in order to create a culture of safety [71]. Only 18.5% of respondents believe that supervisors prioritise speed over safety during difficult periods. This is consistent with the findings of many studies that indicate a weak safety culture. Tobaiqy and Stewart [17] point out that due to a lack of management policies, most medical facilities in Saudi Arabia lack anonymous reporting methods.

According to Alingh [72], HCWs frequently feel uncomfortable raising concerns. Speaking up is significantly influenced by direct supervisors. Research strongly supports the link between PSC and management’s dedication to safety [71]. According to a British study, professional opinions of management and teamwork were linked to significant increases in patient safety as measured by a decrease in complications and mortality rates [73].

Moreover, a healthcare culture that prioritises punishment and blame-shifting over recognising opportunities for learning and improvement may be reflected in the high frequency of blame-shifting in safety-related incident reports. Cooper et al. [74] suggest that it is improbable that analysing incident reports can successfully improve patient safety if a blame-free culture is not established. Consequently, management and supervisor assistance are essential in fostering a safe work environment. Employees may feel more confident to voice safety issues when managers are committed to the process and supervisors are encouraging.

### 4.3. Patient Safety Culture in Dammam and Jeddah

There is limited research comparing the level of patient safety between cities or regions within the same country. Although studies have been conducted addressing PSC within countries [9], little is known about PSC within specific cities or regions of a country.

Generally, there is an increased risk of medical errors and potential patient harm, especially when workloads exceed 40 h per week and include extended overtime shifts of more than 12.5 h per shift. This is supported by the present study’s findings that employee reports of patient safety incidents in Dammam hospitals may be affected by their workload. Furthermore, a study [75] found that working hours are the primary factor contributing to medical errors and an increase in cases that need to be documented, which negatively impacts patient safety.

Moreover, this study’s findings suggest that critical considerations for understanding the patterns of patient safety incident reporting for staff in Jeddah include experience and how it influences the health practitioner’s response to the event, the patient’s reaction to potential outcomes, and prior reporting behaviour. This is in line with a study [76] that found that patients’ reactions to accidents can cause pain, with some patients expressing anger while others seek resolution through the legal system.

This study focused on patient safety incidents at Dammam (*n* = 184) and Jeddah (*n* = 173) hospitals in Saudi Arabia. Models indicate that there are considerable differences between the two cities regarding reporting patient safety incidents. Although response to errors is very important in Jeddah, the culture of communication openness and communication about errors, as well as event reporting, were considerably higher in Dammam. This analysis emphasises the importance of a robust error reporting culture to enhance the reporting of patient safety events [60]. Also, this reveals that authorities should endeavour to develop policies for enhanced PSC, especially by learning from mistakes and avoiding repeating them in the future [23]. It also suggests that reporting practices in Dammam and Jeddah may differ systematically at a regional level. Furthermore, organisational leaders’ support for PS and their policy for continuous improvement was well recognised in Dammam hospitals. By contrast, a study conducted in the Eastern Province by [18] assessed five aspects of PSC: number of incidents, non-punitive response to error, reporting events, patient safety evaluation, and openness of communication. The findings were extremely negative, indicating very low positivity and unsatisfactory results. The study concluded that implementing PSC in healthcare settings requires strong support from organisational leaders.

Compared to the AHRQ, PSC composite measures including handoffs and information exchange, response to error, communication openness, supervisor support, and teamwork were far below the benchmark, while error reporting, event reporting and management’s commitment were close to the benchmark. Finally, in line with the findings of [37] in Riyadh and [18] in KFTH, teamwork was far below the AHRQ-2024 benchmark [19,77].

Furthermore, when interpreting the current results, it is important to consider a few limitations. First, the study’s reliance on online surveys raises the possibility of participation bias. Second, the sample studied was limited, and a larger sample would be desirable in the future for greater representativeness and ensuring the extrapolation of the results. Therefore, generalising the findings of the current study on patient safety culture to the eastern and western regions of Saudi Arabia is not feasible. Also, age and gender, as major demographic characteristics, were not considered in the study and thus represent potential limitations. Finally, the HSOPSC survey study was published in English, indicating a language barrier. Future efforts should focus on developing motivation among HCWs and administrative staff by implementing a condensed version of the questionnaire to improve the response rate.

In terms of recommendations, the primary suggestion is to train professionals consistently and effectively, especially managers and care providers. At an organisational level, it is important to foster a culture that promotes reporting and implements the best practices to cultivate a non-punitive environment. Implementing standardised SBAR (Situation, Background, Assessment, and Recommendation) protocols enhances teamwork and communication [78,79]. When combined with Just Culture algorithms to foster a non-punitive environment, these strategies provide a practical roadmap for translating survey data into measurable safety improvements [80,81,82]. Also, it is recommended to investigate the future directions that focus on “digital safety culture,” addressing risks like unauthorised “shadow AI” and ensuring governance for technology integrated into daily care. Effective interventions prioritise proactive risk monitoring, leadership-driven “just cultures” to encourage reporting, and standardised team training to reduce errors in a complex environment. Finally, all healthcare professionals should be aware that they play a vital role in improving the quality of healthcare services and that no negative consequences will occur due to any reporting.

## 5. Conclusions

Patient safety is a concern for many countries, requiring continuous improvement through the establishment of a safe culture among healthcare providers. This cross-sectional study sheds light on HCWs’ perceptions of PSC in Dammam and Jeddah, Saudi Arabia.

Among the staff members surveyed, physicians and nurses in various positions were the most actively involved in the study, followed by security, transporters, and respiratory therapists. Both error reporting and supervisor support were identified as areas of strength, while potential areas for improvement included management commitment, teamwork, and incident response. Also, teamwork, supervisor support, and hospital management commitment were below AHRQ benchmarks. For improvement, hospitals must replace “blame culture” with active leadership, implement TeamSTEPPS for better teamwork, and ensure supervisors provide structured feedback on all reported incidents. More than 94% of employees rated patient safety positively. Staff in Dammam hospitals are more likely to log patient safety events, including historical reports, depending on their workload and habits. When analysing patient safety incident reporting patterns for staff in Jeddah hospitals, critical considerations include experience, patient interaction, and prior reporting behaviour. Hence, variation in patient safety incident reporting between staff in Dammam and Jeddah hospitals is primarily driven by the interaction of individual habits, workload, and organisational culture.

Comparing the two cities provides authorities with a clearer understanding of vulnerabilities and the importance of protecting HCWs. It is essential to incorporate patient safety training in the learning programmes of hospitals, with an emphasis on SBAR protocols to enhance teamwork and Just Culture algorithms to foster a non-punitive environment. Continuous training, implementation of strategies, effective governance, and an open reporting system are urgently required. These findings can help policymakers and managers enhance patient safety and develop more robust systems to ensure the safety of patients and HCWs in healthcare settings.

## Figures and Tables

**Table 1:** Study participants’ demographic characteristics, job experience, and event patient safety reporting (Dammam = 184 and Jeddah = 173).

Variables	Dammam		Jeddah	
	*n*	%	*n*	%
**Position in this hospital**				
Nurses (Practice, vocational, registered and Assistant)	22	11.9	34	20
Physicians (Assistant, intern, resident)	50	27.1	9	5.2
Dietitian	16	8.7	10	5.8
Pharmacist	0	0	11	6.4
Physical, Occupational or Speech Therapist	6	3.3	11	6.4
Psychologist	21	11.4	7	4
Respiratory Therapist	20	10.9	11	6.4
Technician (e.g., EKG, Lab, Radiology)	1	0.5	3	1.7
Administrative Staff (Supervisor, Manager, Leader, Director)	7	3.8	9	5.2
Food Services	6	3.3	8	4.6
Housekeeping, Environmental Services	2	1.1	0	0
Office workers (IT Services, Secretary, Receptionist)	3	1.6	12	6.9
Security	18	9.8	20	11.6
Transporter	12	6.5	22	12.7
Other (Social workers, facilities)	0	0	6	3.5
**Experience and Number of events reported**				
***How long have you worked in this hospital?***				
1 to 5 years	60	32.6	65	37.6
6 to 10 years	96	52.2	87	50.31
11 or more years	28	15.2	21	12.1
***In this hospital, how long have you worked in your current unit/work area?***				
1 to 5 years	32	17.4	30	17.3
6 to 10 years	113	61.4	89	51.4
11 or more years	39	21.2	54	31.2
***Typically, how many hours per week do you work in this hospital?***				
Less than 30 h per week	27	14.7	21	12.1
30 to 40 h per week	120	65.2	119	68.8
More than 40 h per week	37	20.1	33	19.1
***In your staff position, do you typically have direct interaction or contact with patients?***				
YES, I typically have direct interaction or contact with patients	167	90.8	150	86.7
NO, I typically do NOT have direct interaction or contact with patients	17	9.2	23	13.3
***In the past 12 months, how many patients’ safety events have you reported?***				
None	8	4.3	12	6.9
1 to 2	31	16.8	40	23.1
3 to 5	55	29.9	47	27.2
6 to 10	76	41.3	57	32.9
11 or More	14	7.6	17	9.8

**Table 2:** Staff perceptions of supervisor support, error reporting, event reporting, and hospital management commitment to patient safety.

Statement	Strongly Disagree	Disagree	Neutral	Agree	Strongly Agree	Don’t Know **	Mean (SD)	Level
*Staff Perceptions of Supervisor Support for Patient Safety*								
Supervisor considers staff suggestions for improving patient safety	30 (8.4%)	56 (15.7%)	59 (16.5%)	117 (32.8%)	90 (25.2%)	5 (1.4%)	3.46 (1.32)	High
Supervisor wants us to work faster during busy times (*RI* *)	14 (3.9%)	52 (14.6%)	55 (15.4%)	104 (29.1%)	118 (33.1%)	14 (3.9%)	2.15 (1.25)	Low
Supervisor takes action to address patient safety concerns	11 (3.1%)	34 (9.5%)	64 (17.9%)	126 (35.3%)	112 (31.4%)	10 (2.8%)	3.74 (1.24)	High
**Overall Supervisor Support for Patient Safety**							3.12 (0.91)	Medium
*Participants’ responses about error reporting in their unit/work area*								
We are informed about errors that happen in this unit	8 (2.2%)	18 (5.0%)	60 (16.8%)	140 (39.2%)	119 (33.3%)	12 (3.4%)	3.86 (1.19)	High
When errors happen, we discuss ways to prevent their happening again	7 (2.0%)	66 (18.5%)	82 (23.0%)	87 (24.4%)	103 (28.9%)	12 (3.4%)	3.50 (1.31)	High
We are informed about changes made based on event reports	3 (0.8%)	59 (16.5%)	64 (17.9%)	102 (28.6%)	116 (32.5%)	13 (3.6%)	3.64 (1.31)	High
Staff speak up if they see something negatively affect patient care	7 (2.0%)	44 (12.3%)	74 (20.7%)	97 (27.2%)	126 (35.3%)	9 (2.5%)	3.74 (1.25)	High
When staff see someone with authority doing something unsafe for patients, they speak up	7 (2.0%)	50 (14.0%)	82 (23.0%)	98 (27.5%)	103 (28.9%)	17 (4.8%)	3.53 (1.34)	High
When staff speak up, those with more authority are open to their patient safety concerns	11 (3.1%)	96 (26.9%)	96 (26.9%)	58 (16.2%)	84 (23.5%)	12 (3.4%)	3.20 (1.33)	Medium
Staff are afraid to ask questions when something does not seem right (***RI*** *)	14 (4.2%)	56 (15.7%)	86 (24.1%)	110 (30.8%)	76 (21.3%)	15 (4.2%)	2.38 (1.21)	Low
**Overall, Error Reporting**							3.41 (0.63)	High
*Reporting Patient Safety Events*								
When a mistake is caught and corrected before reaching the patient, how often is this reported?	11 (3.1%)	23 (6.4%)	74 (20.7%)	172 (48.2%)	66 (18.5%)	11 (3.1%)	3.63 (1.14)	High
When a mistake reaches the patient and could have harmed the patient, but did not, how often is this reported?	6 (1.7%)	65 (18.2%)	55 (15.4%)	91 (25.5%)	129 (36.1%)	11 (3.1%)	3.67 (1.34)	High
**Overall Reporting Patient Safety Events**							3.65 (0.98)	High
***Hospital Management Commitment to Patient Safety***								
Actions of hospital management show that PS is a top priority	16 (4.5%)	20 (5.6%)	65 (18.2%)	109 (30.5%)	138 (38.7%)	9 (2.5%)	3.86 (1.26)	High
Hospital management provides adequate resources to improve patient safety	13 (3.6%)	15 (4.2%)	45 (12.6%)	96 (26.9%)	171 (47.9%)	17 (4.8%)	3.97 (1.37)	High
Hospital management seems interested in PS only after an adverse event happens (*RI* *)	25 (7.0%)	101 (28.3%)	79 (22.1%)	84 (23.5%)	52 (14.6%)	16 (4.5%)	2.76 (1.32)	Medium
When transferring patients from one unit to another, important information is often left out (*RI* *)	32 (9.0%)	107 (30.0%)	73 (20.4%)	62 (17.4%)	69 (19.3%)	14 (3.9%)	2.80 (1.39)	Medium
During shift changes, important patient care information is often left out (*RI* *)	34 (9.5%)	90 (25.2%)	68 (19.0%)	78 (21.8%)	70 (19.6%)	17 (4.8%)	2.69 (1.41)	Medium
During shift changes, there is adequate time to exchange all key patient care information	15 (4.2%)	36 (10.1%)	65 (18.2%)	114 (31.9%)	114 (31.9%)	13 (3.6%)	3.66 (1.33)	High
**Overall Hospital Management Commitment to Patient Safety**							3.29 (0.8)	Medium

* RI: the negatively worded items; ** Don’t know: excluded from calculations.

**Table 3:** Association between patient safety rating and the reporting of patient safety events.

Patient Safety Rating	In the Past 12 Months, How Many Patients’ Safety Events Have You Reported? *					Total
	None	1 to 2	3 to 5	6 to 10	11 or More	
Poor	4	4	5	5	4	22
	(18.2%)	(18.2%)	(22.7%)	(22.7%)	(18.2%)	(100.0%)
Good	16	67	97	128	27	335
	(4.8%)	(20.0%)	(29.0%)	(38.2%)	(8.1%)	(100.0%)
Total	20	71	102	133	31	357
	(5.6%)	(19.9%)	(28.6%)	(37.3%)	(8.7%)	(100.0%)
Chi-square = 10.71, *p*-value = 0.030 **						

* *n* (%); ** Significant at 0.05.

**Table 4:** PSC in Dammam and Jeddah cities.

Composite	Region *	Mean (SD)	T	df	*p*-Value
Teamwork	Dammam	3.49 (0.74)	1.991	355	0.047 **
	Jeddah	3.27 (1.26)			
Organisational learning and continuous improvement	Dammam	3.49 (0.73)	1.896	355	0.059
	Jeddah	3.30 (1.15)			
Response to errors	Dammam	2.81 (0.71)	−1.775	355	0.077
	Jeddah	2.96 (0.96)			
Supervisor Support	Dammam	3.23 (0.65)	2.465	355	0.014 **
	Jeddah	3.00 (1.11)			
Communication Openness	Dammam	3.30 (0.48)	2.431	355	0.016 **
	Jeddah	3.12 (0.84)			
Communication about error	Dammam	3.82 (0.54)	3.542	355	<0.001 **
	Jeddah	3.50 (1.09)			
Events Reporting	Dammam	3.86 (0.69)	4.340	355	<0.001 **
	Jeddah	3.43 (1.16)			
Hospital Management Support for Patient Safety	Dammam	3.71 (0.69)	3.816	355	<0.001 **
	Jeddah	3.34 (1.13)			
Handoffs and Information Exchange	Dammam	3.05 (0.83)	−0.012	355	0.990
	Jeddah	3.05 (1.14)			
Patient Safety Rating	Dammam	4.29 (0.62)	4.921	355	<0.001 **
	Jeddah	3.83 (1.11)			
Staffing and Work pace	Dammam	2.86 (0.57)	1.510	355	0.132
	Jeddah	2.74 (0.86)			

* Dammam (*n* = 184) and Jeddah (*n* = 173); ** Significant at 0.05.

## Data Availability

Dataset available on request from the authors.
